# Under-Five Child Poverty and Income Inequality in South Africa: A Multidimensional Perspective at the Lowest Geographic Areas

**DOI:** 10.3390/ijerph22010006

**Published:** 2024-12-24

**Authors:** Jabulani Mathebula, Olufunke Alaba

**Affiliations:** Faculty of Health Sciences, School of Public Health, The University of Cape Town, Cape Town 7925, South Africa; olufunke.alaba@uct.ac.za

**Keywords:** child poverty, multidimensional approach, income inequality, logistic analysis

## Abstract

Under-five child poverty and income inequality are complex socio-economic phenomena that significantly impact the well-being of children worldwide. While there is a growing body of literature addressing child poverty in South Africa, our understanding of settlement discrepancies and factors influencing multidimensional under-five child poverty and income in the country remains limited. This study assesses under-five-specific multidimensional poverty and the determinates of child poverty and inequality in the lowest geographical areas in South Africa. Alkire-Foster’s methodology was applied to construct and estimate the multidimensional poverty index for under-five children across settlement areas. The selected indicators were designed to reflect the unique deprivations and challenges faced by children in this age group. The results showed that there is a significant number of children who are deprived in indicators such as access to ECD (43%), hunger (20%), and education and employment at 69% and 65%, respectively. These are some key variables that require policy interventions to improve the development outcomes of children. The logistic regression results showed that 14 out of the 34 predictors were significant. Inequality was significant and positive at 1%. The findings suggest that more work still needs to be undertaken to improve the living conditions of children, especially in the former homelands, to achieve the goals set in the National Development Plan and Sustainable Development Goals. In the past, non-whites were assigned a homeland according to their ethnicity or a place where national or ethnic identity has formed.

## 1. Introduction

Eleven million children (0–17 years) are poor in South Africa when using an upper bound income poverty line that allows just enough for minimum adequate nutrition and other essentials [1]. The country is one of the wealthiest in Africa, with a gross domestic product (GDP) of ZAR 81,875 per capita in 2020 [2]. South Africa is also one of the countries with the highest level of inequality in the world and a high level of poverty. One out of every two South Africans is poor [3]. Some 30.4 million South Africans lived in poverty in 2015 [3]. The country also has one of the highest Gini coefficients globally, at 0.63, representing an increase since 1994, at the dawn of its democracy, when it was 0.61 [4]. The consequences of the unjust apartheid government worsen the level of poverty and inequality. Poverty and inequality have different effects on adults and children. Children are more likely than adults to live in poor households, and women are more likely than men to live in poor households [5]. This is because poverty has different causes and effects, and the impact of poverty during childhood can have detrimental effects on children that are irreversible [6,7].

Children need enough resources to grow strong and healthy, receive proper education, and live in safe homes to fulfil their potential. Children are said to be living in poverty when they are deprived of these necessities. The term child poverty is used to describe this situation. The United Nations defines child poverty as children who experience and witness deprivation of the material resources required to survive, develop, and thrive, leaving them unable to enjoy their rights, achieve their full potential, or participate as complete and equal members of society. Deprivations are loosely regarded as unsatisfactory and undesirable circumstances, whether material, emotional, physical or behavioural, as recognised by a fair degree of societal consensus [8].

In South Africa, child poverty (among persons under the age of 18) has, however, been declining since the year 2000. It declined from 73% to 60.2% in 2012 [9]. In 2018, 59% of children lived in poverty [10]. Studies on child poverty mainly concentrated on poverty and inequality at the national and provincial levels. Studies that focus on child poverty (age 0–17) at this level include [11,12], which defined children as poor when they belonged to the poorest 40% of households. These studies argued that measuring poverty with a monetary metric technique is not accurate because poverty is multidimensional. The dimensions considered include basic human needs such as food, safe drinking water, sanitation facilities, health, shelter, education, information, and other basic social services [8,13,14]. Studies that applied the multidimensional indicators include [15,16,17,18]. In the case of Noble et al. [19], looked at five domains of deprivation: income and material deprivation, employment deprivation, health deprivation, education deprivation, and living condition deprivation. They found 6 wards in the most deprived 25% for all five domains in the Western Cape Province and 41 wards in the most deprived 25% for all domains in the Eastern Cape Province.

This suggested that there is a dearth of evidence on the under-five multidimensional poverty index at the lowest geographical settlements. Thus, this study explores under-five child poverty in the lowest geographical areas in South Africa and the determinants of child poverty and inequality in various settlement types in South Africa. It is important to understand the status of child poverty and factors leading to child poverty at the lowest geographical level, as the results will enable the government to design targeted policies to improve the nutritional status and living conditions of children.

## 2. Literature Review

Poverty has been generally accepted to mean significant deprivations in well-being [20]. Different dimensions have been employed to understand well-being. For example, reference [21] considered income or consumption as proxies in explaining the term well-being since money can serve as an input to attain any level of utility. Attainment of given dimensions by a household or individuals such as health, education, and nutrition were also used to explain well-being [20]. It was concluded that poverty arises when people lack key capabilities and have inadequate income or education, poor health, insecurity, low self-confidence, or in some way experience powerlessness or the absence of rights such as freedom of speech [21].

Multidimensional approaches to poverty assessment are gaining widespread recognition due to the fact that they incorporate indicators related to basic human needs. The approach reflects a comprehensive understanding of poverty beyond just income measures. Thus, the use of this approach is increasing across developed and developing countries including South Africa.

In South Africa, the provincial landscape is characterised by significant socio-economic and demographic diversity, highlighting a complex issue of circumstances across different regions and geographical areas. Despite this diversity, there has been limited attention directed to children, especially under-five children, within the scope of poverty and inequality discourse. Available studies at the beginning of the millennium focused on the estimation of household poverty and inequality at the provincial and municipal levels [18,19,22]. Provincial indices of multiple deprivation (PIMD) were designed using national data sets such as the census in 2001 [20] with the objective of producing ward-level deprivation indices. The five domains of deprivations considered were income and material deprivation, employment deprivation, health deprivation, education deprivation, and living environment deprivation.

In 2009, improvements were made to the PIMD to produce the South African Index of Multiple Deprivation (SAIMD) using the 2001 census [17]. Five domains of deprivation were also designed for the SAIMD. The PIMD overlooked the differences in population sizes. This meant that provinces with large wards would tend to be under-represented in national indices of deprivation, and pockets of deprivation in larger wards may be ‘diluted’ or hidden by relative non-deprivation in the vicinity [23]. The 2001 Census Enumerations Areas were used to create the lowest geographical areas called data zones. Data zones comprise one or more contiguous EAs that share common characteristics.

After these measurements were developed, they were used to map out the levels of poverty at the municipal level, and the findings showed that income and multidimensional poverty with inequalities vary across municipalities [16]. Municipalities with higher levels of inequality were also found to have higher levels of poverty. Historically disadvantaged areas in terms of low economic activities were found to be experiencing high levels of poverty and inequality.

The community survey of 2007 was used to provide an analysis of child poverty based on income levels. Equivalisation methods were used to consider variations in household sizes and composition [22]. Western Cape and Gauteng had the lowest rate of child poverty at 66%and Eastern Cape and Limpopo had the highest child poverty rates at 88.5%and 88.2% respectively. Municipalities in the Western Cape and Gauteng provinces had the lowest child poverty rates. Municipalities with the highest poverty rates were found in provinces with former homeland areas of Eastern Cape, KwaZulu–Natal, Northwest, and Limpopo provinces. Having relatives in the household, a higher living standard, higher income, good caregiver mental health, and a positive view of the caregiver’s health were factors associated with child well-being. Lack of access to running water and electricity was associated with child well-being. This condition, characterised by severe deprivation of basic human needs, including food, safe drinking water, sanitation facilities, health, shelter, education, and information, depends not only on income but also on access to social services.

In 2015, the Child-Multidimensional Poverty Index (MPI) was developed to explore child poverty and inequality in children between 0 and 17 years. Child-MPI is composed of 18 indicators across four dimensions: education, health, living condition, and economic activity. Data from the 2002 and 2014 General Household Surveys (GHS), published by Statistics South Africa, were used. The data contain information on housing services, social services, household tourism activities, labour markets, and socio-economic information relating to education, living standards, health and other health-related behaviours of the South African population. The assessment was conducted on children between 0 and 17 years of age. MPI is a non-monetary index developed using the Alkire and Foster methodology. The results indicated a reduction in Child MPI over time, from 0.15 in 2002 to 0.09 in 2014. Child poverty also decreased between 2001 and 2011. However, cases of deprivation remained high (over 30%) for some indicators. These findings were further supported by Statistics South Africa, which found that more than 60% of children aged between 0 and 17 years were experiencing multidimensional child poverty. The majority of the children experiencing child poverty were black children (68.3%). Child poverty is more concentrated in rural areas (88.4% than in urban areas (41.3%) [18]. A similar study in China also found that relative poverty is more concentrated in rural areas than in urban areas [24].

Some of the factors that lead to an increase in poverty included larger household size, no employed members in the household, female-headed households, and household heads with low levels of education. Children who grow up in female-headed households are 20 times more likely to experience child poverty than children who grow up in male-headed households and between 60 and 70% of black children experience child poverty [25]. Income inequality further worsens poverty and unemployment. South Africa still has the highest levels of income inequality in the world with a Gini coefficient of 0.67%, followed by Namibia with a Gini coefficient of 0.59. These high inequality levels in South Africa are worsened by the past unjust apartheid policies. Recent literature has shown that since the dawn of its democracy, inequality levels have worsened [26]. Inequality levels are on the rise because firstly, the government finds it difficult to develop strong coalitions to support development strategies, secondly, unequal societies typically experience low growth due to low investor uncertainty, and lastly, unequal societies suffer from high levels of crime and violence [27].

The literature has shown that poverty and inequality studies have been widely conducted in the country. However, these studies have not been focused on children under the age of five. Therefore, (a) this study examined the levels of child poverty among children under the age of five in lower geographic areas, and (b) it investigated the determinants of child poverty.

## 3. Methodology

### 3.1. Data

The study was based on the Living Condition Survey (LCS) 2015. LCS 2015 collected information from 23,380 households. LCS consists of data on household consumption and expenditure [28]. Unlike most available data sets, LCS is representative beyond the provincial boundaries, to the lowest geographical levels such as settlement types. Settlement types are commonly divided into urban and rural areas. Statistics South Africa defines these settlements as urban formal, urban informal, traditional areas, and rural areas. Urban areas are composed of formal urban areas and informal urban areas, while rural areas are composed of traditional and rural areas.

- Urban formal settlements are structured and organised. Land parcels (plots or erven) are formal and permanent structures. A local council or district council controls development in these areas. Services such as water, electricity, and waste removal are provided, and roads are formally planned and maintained by the council. This category includes suburbs and townships.
- Urban informal occurs on land that has not been surveyed or proclaimed as residential, and the structures are usually informal. They are usually found on the outskirts of towns, in pockets of ‘infill’ inside towns, or along railways and roads. Some informal areas are also found in tribal areas (e.g., in Mpumalanga) and in townships.
- Traditional areas (former homelands) were created during the apartheid era to house black populations to prevent them from living in urban areas, for example, Transkei and Venda.
- Rural areas are sparsely populated areas where people farm or depend on natural resources, including dispersed villages and small towns.

LCS is also rich in socio-economic variables to measure Child MPI. The data set consists of 57,684 individuals of which 9743 were children aged 5 and below.

### 3.2. Under-Five Child Multidimensional Poverty Index: Dimensions and Indicators

The study adopted the Alkire and Foster methodology [29], an introduced approach to measuring poverty that uses two cut-offs. Their approach uses a dimension-specific deprivation cut-off, which identifies whether a person is deprived concerning a dimension [30]. A similar technique was used to design the South African Multidimensional Poverty Index (SAMPI), and [31] adopted the same methods to construct the Youth Multidimensional Poverty Index (YMPI). In 2017 Child MPI was developed to measure child MPI in Pretoria [31]. Their Child MPI focused on children aged 0 to 17 years.

Formally, assuming typical indicator deprivation cut-offs are yj, then a child is considered deprived if his/her achievement for an indicator, for example, ‘xj’, is below the deprivation cut-off, i.e., xj < yj. The Child MPI adopts a poverty cut-off of 1 over 3 (33.33%) following the Alkire-Foster MPI, SAMPI, and Youth MPI. Thus, a child is multi-dimensionally poor if he/she has a deprivation score higher than or equal to 1 over 3, i.e., if a child is deprived in a third or more of the weighted deprivations. For children with a deprivation score below the poverty cut-off, even if it is non-zero, it is replaced by a ‘0’. Doing so is referred to as censoring in poverty measurement [31].

However, similar to authors who explored Child MPI in 2017, our indicators were modified for under-five children since children below the age of 5 have different needs as compared to other age groups. The under-five Child MPI comprises 17 indicators across four dimensions: income and material deprivation, health deprivation, education deprivation, and economic activity. In the income and material deprivation dimension, three indicators were introduced: income poverty, household composition, and access to information. On the health deprivation domain, the relevant indicators for under-five children included disability, medical attention, hunger, early childhood development (ECD), limited ECD activities, child registered with the South African Department of Home Affairs, and developmental problems. The four dimensions are equally weighted so that each of them receives a 1 over 4 weight. Indicators within the dimensions are also equally weighted. This shows the relative importance of each dimension and indicator in determining the overall poverty index. Mathematically, the method is expressed as follows:

We denote indicator j weight as $w_{j}$ such that:

$$\sum_{i=j}^{n}w_{j}=1\;\;\;\;\;\;\;\;\;\;\;\;\;\;\;j=\left\{1,2,3,\ldots \ldots n\right\}$$

Each child is assigned a deprivation score according to his/her deprivations in the component indicators. The deprivation score of each child is calculated by taking a weighted sum of the number of deprivations and lies between 0 and 1. The score reaches a maximum of 1 if the child is deprived in all indicators. A child, who is deprived in any indicator receives a score equal to 1, such that:

$$c_{j}=w_{1}D_{1}+w_{2}D_{2}+w_{3}D_{3}+\ldots \ldots \ldots \ldots \ldots w_{n}D_{n}$$

$D_{j}$ = 1 if the child is deprived in indicator j and $D_{j}=0$, while $w_{j}$ is the weight attached to indicator (j) as defined in above equation.

Upper bound poverty lines (UBPL) of ZAR 992.0, produced by Statistic South Africa in 2015, were used to calculate the proportion of children experiencing income poverty. These poverty line is also consistent with the period in which the data were collected [32]. The UBPL is used since it is the highest poverty line. Since children do not earn an income, child poverty is calculated using the incomes of the main household members. Table 1 and Table 2 shows the indicators. 

Table 2 below shows means of variables respectively.

### 3.3. Inequality Index: Gini Coefficient Index

Income inequality measures the extent to which incomes are distributed unevenly across households. Incomes include salaries, wages, bonuses, capital incomes derived from dividends, state pensions and government transfers of the adult household members. The most common measure of inequality is the Gini coefficient index. The Gini coefficient measures the ratio of the area between the Lorenz curve and the line of equality, sometimes referred to as the concentration area [33]. The greater the distance (area) between the line of equality and the Lorenz curve, the more unequal the income distribution is [34]. The Gini coefficient ranges from 0 and 1, where 0 corresponds with perfect equality and 1 corresponds with perfect inequality. As a measure of inequality, it is considered to be correlated with child poverty as these terms are linked [35]. In mathematical terms:

$$\mathrm{GI}=\frac{\mathrm{Area}\;\mathrm{between}\;\mathrm{line}\;\mathrm{of}\;\mathrm{Equality}\;\mathrm{and}\;\mathrm{Lorenz}\;\mathrm{Curve}}{\mathrm{Total}\;\mathrm{area}\;\mathrm{under}\;\mathrm{Line}\;\mathrm{of}\;\mathrm{Equality}}$$

### 3.4. Determinants of Child Poverty: Logistic Regression Model

Logistic regression model was used to assess the determinants of child poverty. This is a binary model used when the dependent variable is made of two options, such as 0 and 1 or yes and no [36]. The logistic regression model is popular because of the logistic formulae. The function provides estimates in the range from 0 to 1 and an appealing S-shaped description of the combined effect of several risk factors for an event. A general logistic regression model is modelled as follows.

$$\log(\frac{p\left(x\right)}{1-p\left(x\right)})=\beta_{0}+x.\beta$$

Solving for *p* yields:

$$p\left(x\right)=\frac{e^{\beta_{0}+x.\beta }}{1+e^{\beta_{0}+x.\beta }}=\frac{1}{1+e^{-(\beta_{0}+x.\beta )}}$$

where *β* and x are logistic intercept and slope. Logistic regression fits *β* and x,the regression coefficients. The model fits well in the current study; child poverty is represented by 1 and 0, with 1 indicating that a child is poor and 0 indicating that a child is not poor.

## 4. Results and Discussion

### 4.1. Raw Headcount or Incidence of Deprivation

#### 4.1.1. Income and Material Deprivation

Table 3, Table 4 and Table 5 show the raw headcount of poverty per dimension or deprivation. Table 3 shows that at the national level, 59% percent of children are living below the upper bound poverty line (UBPL) and they do not have access to information. This is consistent with the findings of [36]; they also found that children have lower access to basic services. 

Table 3 also shows that 89% of children live in a household with more than five people per room; this has implications in terms of resource sharing in the households. Table 3 further shows that children deprived of income were mainly in the traditional areas (51%) and 34% were in urban formal areas. Children under the age of 18 years living in the same household were mainly found in the traditional areas, and at least 5% were in formal rural settlements. Children who were deprived of information were mainly in traditional areas. Lack of access to information transmitted to television or radio has implications for children because this affects their cognitive abilities. Traditional settlements also have the highest proportion of children located 15 min or 200 m away from the primary water source. Black African children were mostly deprived in this dimension, with 93% leading in the income poverty indicator and 86% in the shelter availability indicator. Coloured children are deprived in the access to information indicator (7%) and shelter availability (7%).

#### 4.1.2. Health Deprivation

Table 4 shows the proportion of children deprived in the health indicator. The hunger indicator shows that 20% of children have gone to bed hungry because of insufficient food in the household. In comparison with the national statistics, only 12% of children went to bed without food in 2022; this was 18% lower than reported figures in 2001 [37]. It was also important to assess whether children are registered with the Department of Home Affairs (DHA). Children registered with the DHA can register for child support grants (CSG). The results indicated that 2% of children were registered with the DHA; this indicates that there may be children who are experiencing poverty because they are not registered for CSG. It was also essential to assess whether children are involved in early childhood development (ECD) activities. The indicator of ECD shows that 43% of children were not exposed to ECD activities. At a national level, about 14%of children between 0 and 7 years are not exposed to ECD activities, indicating the importance of disaggregating analyses of ECD at the lowest levels. It was also assessed whether the children had developmental problems. Developmental problems included mental retardation, attention deficit hyperactivity disorder (ADHD), or any other developmental delay. Children who did not receive medical attention because they could not afford it are mainly in the urban formal (48%) and traditional areas (39%). This is possibly because parents with children move to urban areas, mainly big cities, to look for greener pastures. This table also shows that 54% of the children with limited ECD activities live in traditional areas and 42% are found in urban formal areas. In terms of developmental problems, 43% of children with developmental problems live in traditional areas. Table 4 further shows that children experiencing health deprivation are mostly of the black African race.

#### 4.1.3. Years of Education and Employment

Regarding employment and education deprivation, Table 5 show that 69% of children live in households with members who do not have at least five years of education, and 65% of these children live in households where the working-age adults are unemployed. This indicates that many children are living in poverty because their parents or caregivers do not have enough experience and education to secure decent employment. In urban formal areas, 48% and 44% were experiencing education and employment deprivation, and in traditional areas settlement type 43% of children were deprived of education and employment. In terms of race, most of these children are black African children followed by coloured. Eighty-five (85%) and 87% of black African children were deprived in education and employment while 12% and 7% of coloured children were deprived in the same indicators.

### 4.2. Censored Headcount or Incidence of Poverty

As discussed in the previous sections, children are considered poor when deprived in one-third of the weighted indicators. Table 6 shows the overall Child MPI. Children who were deprived in over 70% of the weighted indicators were more severely deprived than children deprived in 40% of the weighted indicators. This table shows that 21% of children were deprived in one-third of the indicators and 56% of children were deprived in over 60% of the indicators. Only 8% of the children were deprived at 70% of the indicators. Thirty-one percent of children who were deprived in one-third of the indicators were staying on urban formal areas and 23% in urban informal areas. Fifty-one (51%) of the children in urban formal were deprived in over 60% of the indicators. In terms of the settlement types, 51% of children were deprived in over in 60% of the indicators in urban and rural formal areas. In the urban informal area 61% of children were deprived in over 60% of the weighted indicators. Black African children also led the category of children deprived in over 60% of the weighted indicators at 58% followed by coloured children. The less deprived children were white children at 24%. One of the explanations for this is that white Africans mainly stay in well developed areas, mostly urban formal areas where they have well developed systems. This settlement areas were mainly developed by the apartheid government. 

**Figure 1:**
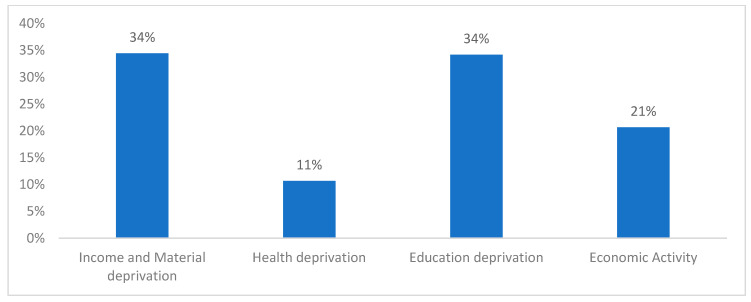
Child MPI composition.

Figure 1 shows that income and material deprivation and education deprivation contributed 34% each to the Child MPI. Health and economic deprivation contributed 11% and 21%, respectively.

Table 7 shows the income inequality by settlement type. There is high income inequality in urban formal areas and urban informal areas at 68% and 57%, respectively. The inequality level in South Africa was at 71% at the national level when using the weighted household income. This is higher than the 61% national-level inequality reported by the World Bank in 2018. The inequality level closest to 71% was reported by [29] at 70%.

Table 8 presents the association of Child MPI and household inequality at the settlement type level. Measures of association show a significant relationship between the two variables. The relationship between Child MPI and the Gini coefficient was highest in urban formal areas with a Gini coefficient of 68%. At the 50th Child MPI, the correlation between Gini and Child MPI was highest in urban formal areas at 74%. At the 70th Child MPI, the relationship between the Child MPI and Gini was highest in the traditional areas at 52%. This indicates direct poverty and household incomes.

### 4.3. Multivariate Regression Results

According to the framework of the UN Convention on the Rights of the Child, articles 27 and 28 food, housing, shelter, and sanitation are important for an adequate standard of living. In this study, children living in households that lack access to information, a good water source, sanitation facilities, shelter facilities, and other basics were associated with multidimensional poverty. Table 9 presents the estimated results for the logistic regression model. Of the 34 variables included in the model, 14 were significant at various significance levels. Additionally, all the significant variables were positively associated with multidimensional poverty except for child age and household composition. Since the dependent variable was coded as 1 if the child is experiencing multidimensional poverty, the positive coefficient indicates that the child is more likely to be poor and vice versa. Income poverty variable was found to be positive significant at 1% indicating that children living in a household with incomes below the poverty level are more likely to experience multidimensional poverty. This was confirmed by [38], they found that the higher the income of the household head, the less likely a child is to be poor

The age of the household head was positive and significant at 10%, indicating that the older the household head, the less likely a child is to be poor. The variable household composition was negative and significant at 1%, indicating that if there is more than one child in the household under the age of five years, child poverty increases. Additionally, [38] found that the higher the number of members in the household, the higher the probability that the children in those households were poor. Access to information was positive and significant at 1%, indicating that children who have access to television or radio are less likely to be deprived of information to enhance their cognitive ability. Access to water is positive and significant at 1%, indicating that children living in households with access to water more than 15 min away have a high chance of living in poverty. Shelter deprivation was positive and significant at 1%; this was unexpected and means that when there are more than five people per room in the household, children experience poverty. The variable for access to basic services was positive and significant indicating that children who live 20 km or more from a preschool or 50 km or more from any medical facility are more likely to experience child poverty.

The ECD variable’s coefficient was positive and significant at 1%. This indicates that children who are not exposed to early childhood activities are more likely to be poor. The variable limited access to ECD activities was positive and significant at 10%. Developmental problems were also found to be positive and significant at 10%. This indicates that children suffering from mental retardation, attention deficit hyperactivity disorder (ADHD), and any other developmental delays are more likely to be poor when children are deprived in all the weighted indicators. The variable on registration with the Department of Home Affairs (DHA) was positive and significant at 1%, indicating that it is important for children to register with the DHA. Children who are registered with DOH are able to apply for child support grants. Socio-economic characteristics of the household, such as employment and education level of household head, are statistically significant with child multidimensional poverty. The results show that children living in households where no adult is employed are more likely to experience higher levels of poverty compared to their counterparts. This is also similar to the findings of [38,39], that children living in households where members of the households are employed are less likely to experience poverty. 

Gini coefficient results at the settlement type level were included in the model to test their influence on child poverty. However, it was not significant. To assess spatial variation, location variables were included, and these are provinces and settlement types. The only province which was significant as a variable was North West.

## 5. Conclusions and Recommendations

The study focused on assessing child poverty and inequality in South Africa among children younger than five years. The study also focused on the contribution of each weighted dimension to the under-five Child MPI. The study emphasises that there are more indicators that can help understand child poverty in children aged five and below. Hence, new indicators such as those focusing on children who are not receiving care dependency grants, children who are not receiving medical attention because of high costs, going to bed hungry, children not receiving ECD, and children not registered with the Department of Home Affairs were introduced in the analysis. Other indicators include children who do not have access to water, television, or radio. 

The results show that one-third of children’s MPI poverty is concentrated in urban formal (31%) and urban informal (23%) areas. These areas also had the highest proportion of children deprived in the 60th percentile with 61% and 51%, respectively. However, at the 70th percentile, traditional areas had the highest level of Child MPI (15%). This means that the children are severely deprived. Urban formal and urban informal are the settlements with highest levels of inequality at 68% and 57%, respectively. This finding confirms that there is a significant relationship between poverty and income inequality. The implications of the results is that there’s a significant number of children who are deprived in key indicators and child poverty levels differ between settlement types. These key indicators include access to ECD, television, or radio for their mental development and being registered with the DHA to access basic services such as social grants and education.

The government has made progress in reducing child poverty and household income inequality, but more work still needs to be done, especially in the former homeland provinces to achieve the goals set out in the National Development Plan and Sustainable Development Goals. To address child poverty at the lowest geographical level, the government, civil sector organisations, and their partners should develop and prioritise policies which are focused on improving child development outcomes. Below are suggested recommendations:

- Recommendation 1: At least one member of the household should be employed or earning an income, which could be through government grants. This is because it was observed that one of the key variables which causes child poverty is lack of income. Reliance on child support grants does not necessarily help in terms of improving the living conditions in the household.
- Recommendation 2: Policies that focus on improving access to information for children aged five and below should be designed. Children should have access to television, radio, and toys etc. which can help children in terms of improving their vocabulary and their cognitive abilities.
- Recommendation 3: Improve access to ECD for children aged five and below. Effective ECD centres helps with improving child concentration, patience and teamwork, encouraging holistic development and in terms of open child to learning. The government, mainly local government, should support ECD centres at the local level.
- Recommendation 4: The government should provide free assistance to children with developmental challenges such as ADHD. The results indicated that children with developmental challenges are struggling to access medical care. The government increases access to these for these children by identifying struggling households referred to as indigent households. Government servants such as councillors can help in terms of identifying households with children who are facing developmental challenges.

A few limitations of the approach may need to be highlighted for future research and extension. There are limited data sources to conduct a study of this nature. Statistics South Africa and other data-collecting agencies should collect data on all children of different age groups at the lowest level possible. This will help in terms of understanding the conditions of children and in designing specific policies to address those challenges at the lowest geographic level. Other key variables that are important to understanding the condition of children that were not included in LCS include information on safety and violence, child mortality, and anthropometric data. Anthropometric data and child immunization data are important in creating a robust Child MPI.

## Figures and Tables

**Table 1:** Child Deprivation Index and variable.

*Deprivation*	*Indicators*	*Definition of Indicators (Deprived If)*
**Income and material deprivation (1 over 4).**	Income poverty (1 over 28).	A child is living below the upper bound poverty line (UBPL).
	Household composition (1 over 28).	The number of children living in a household under the age of 5.
	Source of information (1 over 28).	The number of children under 5 years of age living in households with neither TV nor radio.
	Access to water (1 over 28).	A child who lived in households where the nearest source of water was more than 15 min away.
	Access to sanitation facilities (1 over 28).	Children who had no access to a toilet of any kind in the vicinity of their dwelling, i.e., no private or communal toilets.
	Access to shelter deprivation (1 over 28).	A child living in a household with more than five people per room.
	Access to basic services (1 over 28).	Children living 20 km or more from pre-school or 50 km or more from any medical facility with doctors.
**Health deprivation (1 over 4).**	Disability (1 over 32).	Disabled children aged 0–5 years who are not on Care Dependency Grant (CDG).
	Medical attention (1 over 32).	The child did not receive medical attention because it was too expensive.
	Hunger (1 over 32).	A young child aged 0–5 years, in a household has to go to bed hungry because of insufficiency of food in the house.
	Ill health (1 over 32).	Young children aged 0–5 years who are ill cannot seek medical care due to inability to pay for health care services, distance to health care facilities and other socio-economic reasons.
	Early childhood development (1 over 32).	Young children aged 0–5 years are not exposed to ECD. ECD refers to the emotional, cognitive, sensory, spiritual, moral, physical, social, and communication development of a child.
	Limited ECD activities (1 over 32).	Young children aged 0–5 years are limited in their daily activities at home, work, and school because of long-term physical, sensory, hearing, intellectual, or psychological conditions.
	A child registered with the South African Department of Home Affairs (1 over 32).	Young children aged 0–5 years not registered with the South African Department of Home Affairs.
	Developmental problems (1 over 32).	Young children aged 0–5 years suffering from mental retardation, attention deficit hyperactivity disorder (ADHD) and any other developmental delay.
**Education deprivation (1 over 4).**	Years of schooling (1 over 4).	A child lives in a household where no member has at least five years of education.
**Economic activity (1 over 4).**	Unemployment (1 over 4).	Young children aged 0–5 years are living in a household where no working-age adults are employed, and no member of the household is on any social grant.

**Table 2:** Mean of the variables.

*Variable*	*Variable Definition*	*Mean (Average)*
Age of the child	Age of children in the study (continuous)	3.04 years
Sex of a child	Dummy (1 if male, 0 otherwise)	(Male = 4921; Female = 4822)
**Settlement type**	Categorical variable	9743
Urban formal		4296
Urban informal		611
Traditional area		4486
Rural formal		350
**Province**	Categorical variable	9743
Western Cape		927
Eastern Cape		1314
Northern Cape		536
Free State		817
KwaZulu–Natal		1729
North West		802
Gauteng		1086
Mpumalanga		1086
Limpopo		1446
Age of household head	Continuous variable (Age of household head)	50.05 years
Sex of household head	Dummy (1 if male, 0 otherwise)	Female = 5255; Male = 4488)
Marital status of household head	Dummy (1 if married, 0 otherwise)	Married = 199; Not married = 9544
Income poverty	Continuous (Children living below the UBPL)	Deprived = 6182; Not deprived = 3561
Household composition	Dummy (More than five children under the age of five live in the household; 0 = otherwise)	Deprived = 9669; Not deprived = 74
Access to information	Dummy (1 = Children do not have access to radio or TV, 0 = otherwise)	Deprived = 6182; Not deprived = 3561
Water access	Dummy (1 = Children live in a household where the nearest source of water is more than 15 min away, 0 = otherwise)	Deprived = 1574; Not deprived = 8169
Sanitation facilities	Dummy (1 = Children have no access to toilets, 0 = otherwise)	Deprived = 9430; Not deprived = 313
Shelter deprivation	Dummy (1 = Children live in a household with more than five people per room, 0 = otherwise)	Deprived = 8855; Not deprived = 888
Access to basic services	Dummy (1 = Children live 20 km or more from pre-school or 50 km or more from any medical facility with doctors, 0 = otherwise)	Deprived = 389; Not deprived = 9354
Disability	Dummy (1 = Disabled children not on CDG, 0 = otherwise)	Deprived = 9733; Not deprived = 10
Hunger	Dummy (1 = Children slept hungry because there was insufficient food in the house, 0 = otherwise)	Deprived = 1945; Not deprived = 7798
Medical attention	Dummy (1 = Children not receiving medical attention because it is too expensive, 0 = otherwise)	Deprived = 256; Not deprived = 9487
Ill health	Dummy (1 = Children not receiving medical attention because of distance, payment, and other reasons, 0 = otherwise)	Deprived = 1051; Not deprived = 8692
Children registered with Department of Home Affairs	Children not registered with the Department of Home Affairs	Deprived = 9554; Not deprived = 189
Early childhood development	Dummy (1 = Young children not exposed to ECD activities, 0 = otherwise)	Deprived = 4166; Not deprived = 5577
Developmental problems	Dummy (1 = Children suffering from developmental delay, 0 = otherwise)	Deprived = 43; Not deprived = 9700
Limited ECD activities	Dummy (1 = Children limited in their daily activities due to long-term physical condition; 0 = otherwise)	Deprived = 9699; Not deprived = 44
Employment	Dummy (1 = Children live in a household where no adult member is employed, 0 = otherwise)	Deprived = 8735; Not deprived = 1008
Years of education	Dummy (1 = Children live in a household where no member has at least five years of education, 0 = otherwise)	Deprived = 6916; Not deprived = 2827
Inequality level	Income inequality at the settlement type level	57,684 Households

**Table 3:** Income and material deprivation.

*Indicators*	*Income Poverty*	*Household Composition*	*Access to Information*	*Access to Water*	*Sanitation Facilities*	*Shelter Availability*	*Access to Basic Services*
National	63%	99%	59%	16%	3%	89%	3%
Urban formal	34%	47%	38%	4%	7%	47%	48%
Urban informal	9%	9%	10%	5%	5%	9%	8%
Traditional area	51%	39%	46%	84%	71%	39%	39%
Rural formal	5%	5%	6%	7%	17%	5%	5%
**Population group**							
Black African	93%	87%	91%	99%	97%	86%	91%
Coloured	5%	7%	7%	0%	3%	7%	4%
Indian/Asian	1%	2%	0%	0%	0%	2%	0%
White	1%	5%	2%	0%	0%	5%	5%

**Table 4:** Health deprivation.

*Indicators*	Disability	Hunger	Medical Attention	Ill Health	Child Registered with DHA	Early Childhood Development	Developmental Problems	Limited ECD Activities
National	0.12%	20%	3%	11%	2%	43%	0.44%	0.36%
Urban formal	31%	36%	47%	49%	41%	35%	40%	42%
Urban informal	19%	11%	15%	8%	17%	8%	15%	4%
Traditional area	51%	47%	29%	37%	40%	51%	43%	54%
Rural formal	0%	7%	9%	6%	3%	5%	2%	0%
**Population group**								
Black African	100%	95%	89%	89%	97%	89%	91%	96%
Coloured	0%	4%	3%	4%	2%	7%	4%	4%
Indian/Asian	0%	0%	1%	1%	1%	2%	0%	0%
White	0%	1%	7%	6%	0%	2%	5%	0%

**Table 5:** Education and employment deprivation.

*Indicators*	*Education Deprivation*	*Employment Deprivation*
National	69%	65%
Urban formal	48%	44%
Urban informal	6%	8%
Traditional area	43%	43%
Rural formal	3%	5%
**Population group**		
Black African	85%	87%
Coloured	12%	7%
Indian/Asian	1%	2%
White	2%	5%

**Table 6:** Child MPI.

Child MPI	33.33+	40+	50+	60+	70+
National	21%	7%	8%	56%	8%
Urban formal	31%	4%	13%	51%	1%
Urban informal	23%	8%	5%	61%	2%
Traditional area	11%	9%	3%	62%	15%
Rural formal	15%	13%	8%	51%	13%
Black African	19%	8%	7%	58%	9%
Coloured	32%	7%	9%	51%	1%
Indian/Asian	46%	4%	14%	36%	0%
White	46%	1%	28%	24%	0%

**Table 7:** Income inequality by settlement types.

*Settlement Types*	*%*
Urban formal	68%
Urban informal	57%
Traditional area	52%
Rural formal	52%

**Table 8:** Child MPI by household inequality at the settlement type level (n = 9743).

*Child MPI*	*33.33+*	*40+*	*50+*	*60+*	*70+*	*Total*
Traditional area	23%	56%	15%	47%	86%	44%
Rural area	4%	9%	5%	5%	9%	5%
Urban informal	10%	10%	6%	9%	3%	9%
Urban formal	63%	25%	74%	38%	3%	43%

Pearson chi-square = 0.000 (Significance level of the relationship).

**Table 9:** Logistic regression results.

	*Coefficient*	*Standard Deviation*	*95% Confidence Interval*
Age of household head	0.01	0.011	(−0.0077962; 0.0339439)
Sex of household head	0.134	0.271	(−0.3964883; 0.6639215)
Age of child	−0.307 ***	0.096	(−0.4951187; −0.1192902)
Sex of child	0.090	0.246	(−0.3925541; 0.5727383)
Marital status	−0.304	0.717	(−1.708634; 1.100245)
Income poverty	6.936 ***	0.457	(6.040428; 7.830867)
Household composition	−2.229 ***	0.802	(−3.800183; −0.6583152)
*Access to information*	7.013 ***	0.431	(6.169417; 7.85742)
Water access	4.689 ***	0.586	(3.540656; 5.837389)
Sanitation facilities	3.450 ***	0.923	(1.641543; 5.25771)
Shelter deprivation	6.154 ***	0.538	(5.100371; 7.207358)
Access to basic services	3.270 ***	0.807	(1.687284; 4.851889)
Disability	0.237	2.442	(−4.549058; 5.023834)
Hunger	0.343	0.440	(−0.5202875; 1.205447)
Medical attention	0.140	0.664	(−1.161769; 1.442432)
Ill health	0.043	0.376	(−0.6950456; 0.7805468)
Child registered with Department of Home Affairs	2.888 ***	0.767	(1.38542; 4.390963)
Early childhood development	5.577 ***	0.375	(4.841941; 6.31264)
Developmental problems	7.702 *	4.570	(−1.255798; 16.65996)
Limited ECD activities	3.836	15.504	(−26.55169; 34.22321)
Years of education	15.915 ***	0.903	(14.1454; 17.6838)
Employment	15.782 ***	0.839	(14.13669; 17.42656)
Gini-co-efficient	53.123	711.691	(−1341.765; 1448.011)
Western Cape	(Ref)		
Eastern Cape	−0.178	0.551	(−1.258812; 0.9021694)
Northern Cape	0.240	0.557	(−0.8528052; 1.332418)
Free State	0.315	0.538	(−0.739426; 1.369422)
KwaZulu–Natal	−0.836	0.551	(−1.91617; 0.2450988)
North West	1.137 **	0.613	(−0.0641365; 2.337755)
Gauteng	0.139	0.458	(−0.7592217; 1.037162)
Mpumalanga	0.251	0.559	(−0.8455702; 1.346829)
Limpopo	−0.040	0.616	(−1.246822; 1.167241)
Urban formal	(Ref)		
Urban informal	5.351	76.866	(−145.3035; 156.0055)
Traditional area	8.736	113.159	(−213.0526; 230.5238)
Rural formal	8.965	108.887	(−204.4503; 222.3802)
Constant	−58.856	485.379	(−1010.18; 892.4689)

Notes ***, **, and * represent “*p*-values” 1%, 5% and 10% significant levels, respectively.

## Data Availability

Publicly available dataset wase used. The data is called Living Condition Survey (LCS 2015), produced by Statistics South Africa.
